# A two-wheeled machine with a handling mechanism in two different directions

**DOI:** 10.1186/s40638-016-0049-8

**Published:** 2016-10-13

**Authors:** Khaled M. Goher

**Affiliations:** Department of Informatics and Enabling Technologies, Lincoln University, Lincoln, New Zealand

**Keywords:** Lagrangian formulation, IP new configuration, Inverted pendulum, Two-wheeled vehicle, Payload handling, PID control

## Abstract

Despite the fact that there are various configurations of self-balanced two-wheeled machines (TWMs), the workspace of such systems is restricted by their current configurations and designs. In this work, the dynamic analysis of a novel configuration of TWMs is introduced that enables handling a payload attached to the intermediate body (IB) in two mutually perpendicular directions. This configuration will enlarge the workspace of the vehicle and increase its flexibility in material handling, objects assembly and similar industrial and service robot applications. The proposed configuration gains advantages of the design of serial arms while occupying a minimum space which is unique feature of TWMs. The proposed machine has five degrees of freedoms (DOFs) that can be useful for industrial applications such as pick and place, material handling and packaging. This machine will provide an advantage over other TWMs in terms of the wider workspace and the increased flexibility in service and industrial applications. Furthermore, the proposed design will add additional challenge of controlling the system to compensate for the change of the location of the COM due to performing tasks of handling in multiple directions.

## Background

Two-wheeled robots are based on the idea of the inverted pendulum (IP) system. It is a well-identified benchmark problem that provides many challenges to control design. The IP system is nonlinear, unstable, nonminimum phase and under-actuated. The inverted pendulum problem is one of the most well-known conventional problems in control theory and has been investigated extensively in the literature.

Motion control and stability analysis of a two-wheeled vehicle (TWV) are presented by Ren et al. [29] where a self-tuning PID control strategy, based on a reduced model, is proposed for implementing a motion control system that stabilizes the TWV and follows the desired motion commands. Chan et al. [5] explored the common methods which have been investigated and the controllers which have been used of two-wheeled robots on different types of terrains. Shojaei et al. [30] proposed an adaptive robust tracking controller to cope with both parametric and nonparametric uncertainties of the system occurred due to the integrated kinematic and dynamic trajectory tracking control problem wheeled mobile robots. Deng et al. [11] designed controller based on Lyapunov function candidate and considered virtual forces information including detouring force. Guo et al. [15] design a sliding mode controller for wheeled IP. Li and Kang [23] used the technique of dynamic coupling switching control for a wheeled manipulator. Actuator faults and abnormalities in operation in a two-wheeled IP system has been investigated by Tsa et al. [33].

Investigating the parametric and functional uncertainties has been also considered in the literature; Li et al. [20–22] considered the dynamic balance and motion control based on least squares support vector machine for wheeled inverted pendulums (WIP) subjected to dynamics uncertainties. Control algorithms, using Lyapunov synthesis, with the advantage of LS-SVM combined with online parameters estimation strategy have been proposed. Based on this approach, the outputs of the system proved to be able to track the given bounded reference signals within a small neighbourhood of zero as well as guarantee semi-global uniform boundedness of all the closed-loop signals. An intelligent backstepping tracking control system is proposed by Chiu et al. [6, 7] for WIPs with unknown system dynamics and external disturbance. An adaptive output recurrent cerebellar model articulation (AORCMAC) is used to copy an ideal backstepping control (IBC), and a compensated controller is designed to compensate for difference between the IBC law and AORCMAC. In a further work by Chiu et al. [6, 7], a novel model-free intelligent controller to control WIPs has been developed. An adaptive output recurrent cerebellar model articulation controller (AORCMAC) for angle and position control of the WIP without model information has been developed. Lee et al. [19] carried out a historical evolution of IP systems for several designs. Ghaffari et al. [12] used Kane’s and Lagrangian dynamic formulation methods to drive the dynamic model of a self-balancing two-wheeled robot. Ping et al. [26, 27] reviewed various methods of driving the dynamic model and control techniques used for two-wheeled robots.

Cui et al. [9] designed a state feedback control for a wheeled IP, and then backstepping-based adaptive control is designed for output tracking of the system. Brisilla and Sankaranarayanan [4] proposed a nonlinear control strategy for a mobile IP without internal switching between controllers. Chinnadurai et al. [8] used internet on a chip controller to design a two-wheel robot using the principle of curvature technique. Dai et al. [10] designed a method based on friction compensation for two-wheeled IP. Raffo et al. [28] designed H∞ nonlinear controller to stabilize and control two-wheeled machine under the presence of exogenous disturbances. Sun and Li [32] used adaptive neural control and extreme learning machine (ELMs) to develop and implement on two-wheeled human transportation system. A novel control scheme is developed based on a single-hidden layer feedforward network approximation capability of combing ELMs to capture vehicle dynamics. Yue et al. [34] investigated error data-based trajectory planner and indirect adaptive fuzzy control with the application on two-wheeled IP using indirect adaptive fuzzy and sliding mode control approaches, Lyapunov theory and LaSalle’s invariance theorem. Yue et al. [35] designed a composite control approach for balancing and trajectory tracking of two-wheeled IP vehicle using adaptive sliding mode, fuzzy-based control and adaptive mechanism.

### Principle of two-wheeled IP with an extended rod

**Fig. 1:**
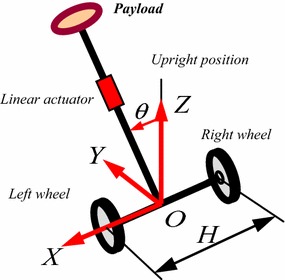
Single IP with an extended rod

**Fig. 2:**
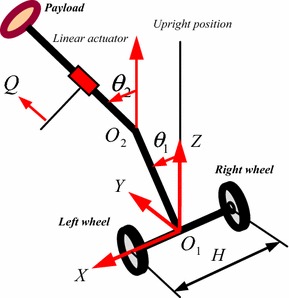
Double IP with an extended rod

The principle of two-wheeled IP with an extended intermediate body (IB) has been first introduced by Goher and Tokhi [13, 14] where a new configuration of wheeled robotic machines (WRM) is developed and equipped with a linear actuator, as shown in Fig. 1, to activate a payload and to lift it to different levels. Although the developed configuration added additional DOF through the linear actuator attached to IB, the workspace has been extended only in one single vertical direction by extending the IB. In a further work to increase the workspace and the TWM flexibility, Goher [2, 14] developed a two-wheeled IP where an additional link is added, shown in Fig. 2, to end with a five DOFs double IP system with an extended rod.

**Fig. 3:**
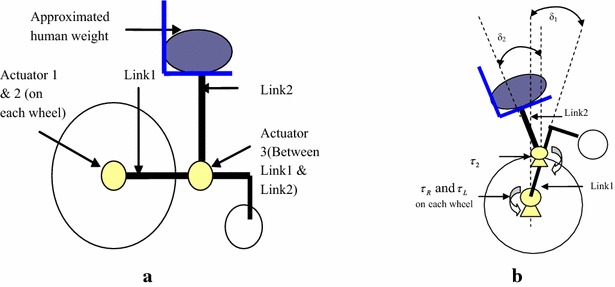
Schematic diagram of wheelchair transfer. **a** Wheelchair (before lifting). **b** Wheelchair (during lifting)

The application of the double IP with an extended rod configuration has been utilized to simulate an important scenario of wheelchair transfer to stand on two wheels, shown in Fig. 3a, b, as presented by Ahmad et al. [1]. In this research, the authors also used a linear actuator attached along link 2 to further lift the chair and the person to a further specified height.

**Fig. 4:**
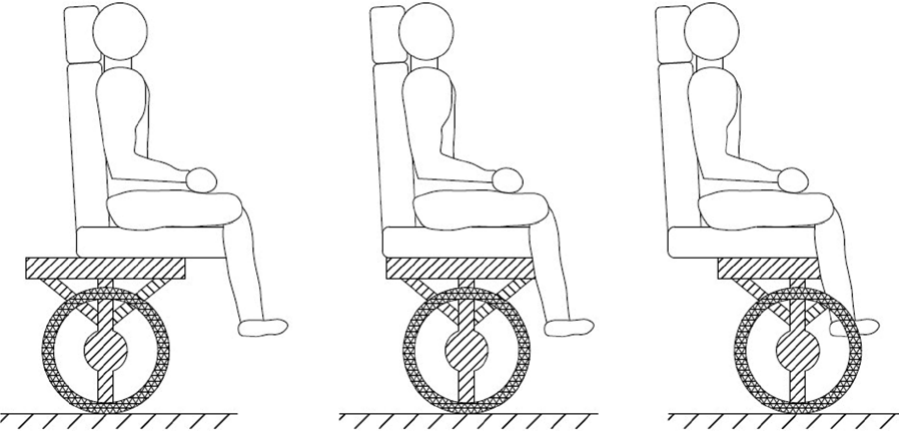
UW-Car with an adjustable seat in horizontal motion

**Fig. 5:**
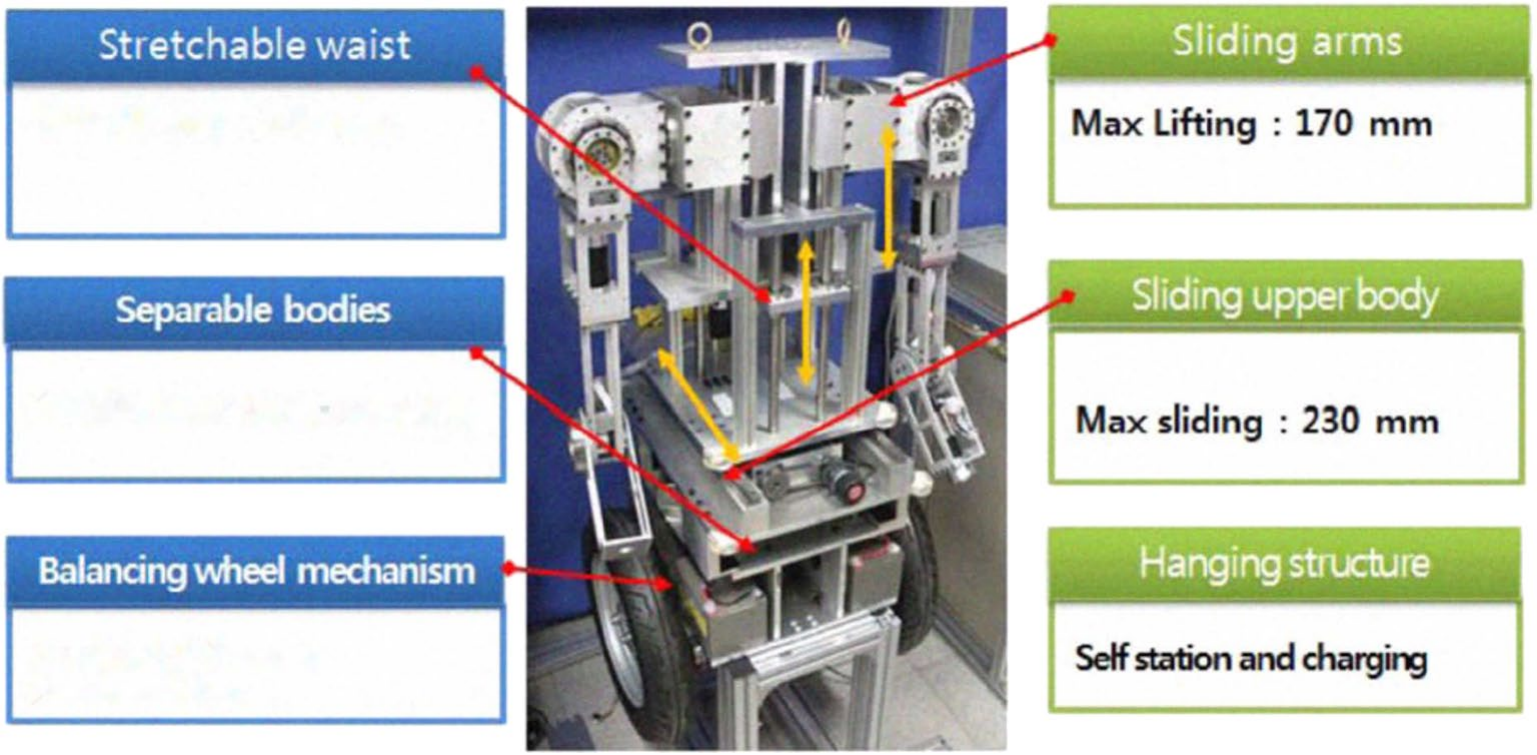
Korean service robot (KOBOKER)

A two-wheeled robot, TransBOT, is developed by Lee and Jung [18]. TransBOT has two driving modes, driving mode and a balancing mode that mimics the IP concept by lifting up the front casters. The developed prototype is similar to the PUMA human transporter, and its working principle mainly relies on stabilizing payload in one single direction. In the work done by Huang et al. [16], a vehicle called UW-Car, with a schematic diagram shown in Fig. 4, is developed where a movable seat is driven by a linear motor along a straight horizontal direction. A control algorithm is developed and implemented both in MATLAB simulation environment and on real experimental of the developed prototype. Although this work considered an adjustable position of the car seat in horizontal direction, the motion of the seat in a vertical direction has not been considered. Furthermore, Bae and Jung [3] developed service robots, KOBOKER shown in Fig. 5, that is able to self-balance using up and down sliding mechanism that activates two arms in order to perform tasks on the floor. The design of KOBOKER allows handling of objects in two different directions and is equipped with two serial arms to handle different tasks as per specified.

Despite the above-mentioned contributions in terms of developing new configurations of TWMs, the dynamic analysis of TWMs with mass balancer in two different directions has not been given too much interest in the literature. A dynamic model of this new configuration will have the potential to form the basis for new applications and exploration of many features of the system as well as the possibility to investigate the impact of various characteristics. In this current work, a novel configuration of TWMs is introduced that enables handling payload attached to the IB in two mutually perpendicular directions. This will allow extension of the workspace of the vehicle and to increase its flexibility in various applications including: material handling, objects assembly and similar industrial and service robots application. The proposed configuration, with similar concept as KOBOKER [3], gains both advantages of serial robots and TWMs that occupy a minimum space due to working on two wheels only.

A model of this new configuration will have the potential to form the basis for new applications and exploration of many features of the system as well as the possibility to investigate the impact of various characteristics. The novel configuration of the vehicle with five DOFs provides the vehicle with an ability to handle objects in two mutually perpendicular directions. This is achieved by either a dual-axes linear actuator or two different actuators that will be able to extend the intermediate body (IB) of the vehicle in two different directions. In this work, five decoupled feedback control loops have been used throughout this work. The developed control strategy, based on loops decoupling, ensures separation of the dynamics due to the high frequency range (tilt angle) from the dynamics of low frequency range (motion of the intermediate body). Various simulation exercises have been considered to test the robustness of the developed control scheme. Even with complicated scenarios of changing the COMs simultaneously in two different directions, the control strategy was able to cope well with such variations. Internal system dynamics have been considered to test the robustness on the control approach. Huang et al. [16] used on the other hand LQR and sliding mode controllers to control the velocity and braking of a two-wheeled vehicle. Though the system was developed by Bae and Jung [3], no control has been considered in their work.

The rest of the paper is organized as follows: “Introduction” summarizes relevant contributions in TWMs and the associated control strategies. “System description” section describes the system with the proposed configuration, explanation of the system DOFs and detailed description of a picking and placing scenario while handling an object in a confined space. The mathematical model of the system state space is derived in “Mathematical modelling” section, and a linearized state space model is derived in “State space modelling” section. PID control scheme is designed in “Numerical simulation” section and implemented on the system model based on a set of numerical parameters. Various simulation exercises are used for the numerical validation including either sequentially or simultaneously change of COM of the vehicle in two different directions. Finally, the paper is concluded in “Conclusion” where the work contributions are highlighted and a set of recommendations are formulated for potential future work.

## System description

**Fig. 6:**
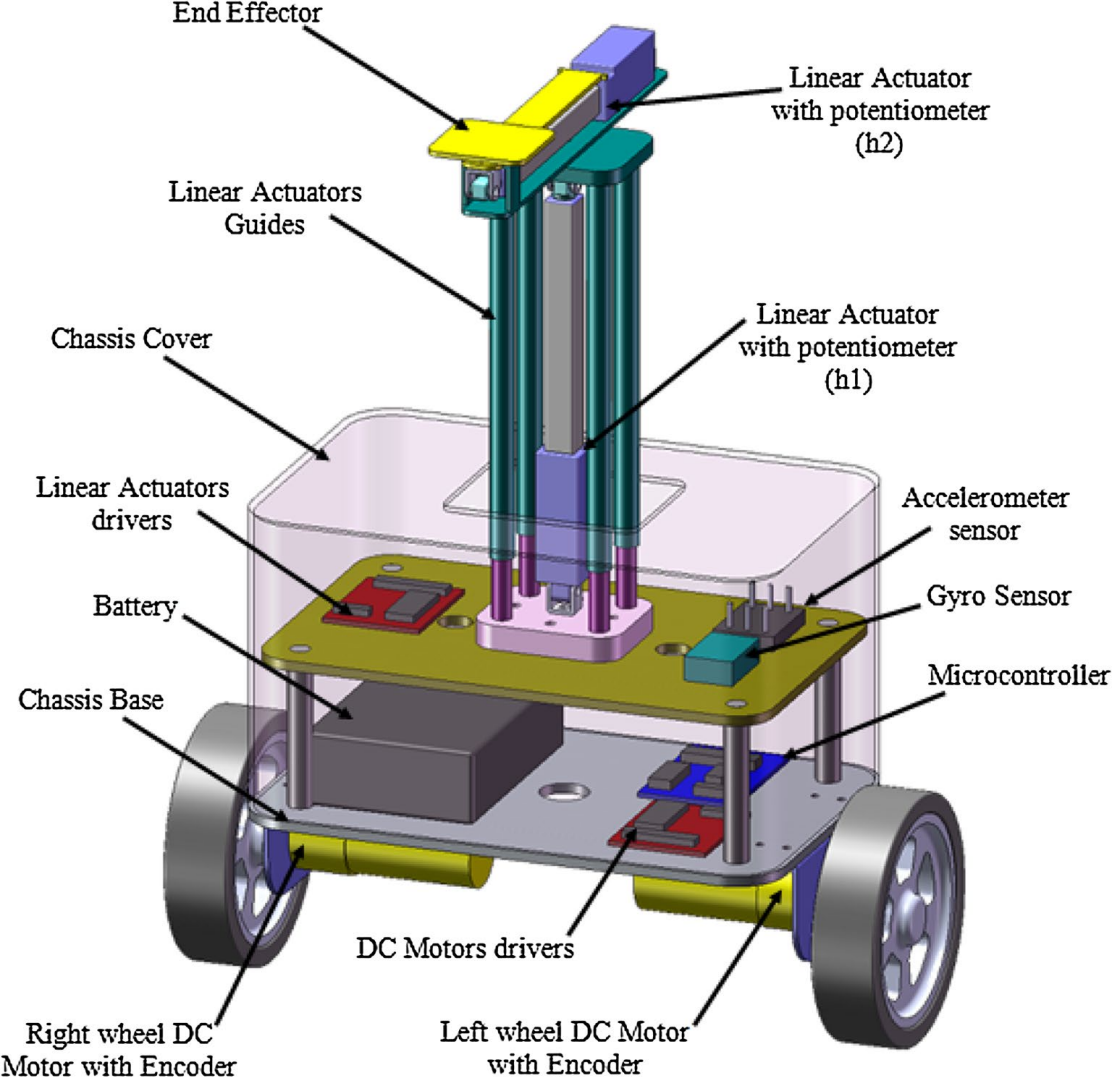
Schematic diagram of system in Solidworks

**Fig. 7:**
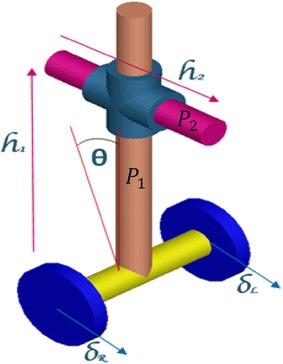
ADAMS/View model of the 5D-TWRM

The proposed TWRM has five DOFs as shown in Figs. 6 and 7 where Solidworks^®^ and ADAMS MSC^®^ are used to generate the design. The proposed vehicle consists of a chassis with centre of gravity at point $P_{1}$ and the mass of the linear actuators with centre of gravity at point $P_{2}$. The coordinates of points $P_{1}$ and $P_{2}$ will change if the robot moves away from its initial location along X axis. These variables fully describe the dynamics of the five DOFs system. The two-wheeled robot is controlled by applying a torque $\tau_{\text{R}}$ and $\tau_{\text{L}}$ to the right and left wheels, respectively. This torque is contributed by the motors attached to each wheel. Other inputs that enable the control system to keep the robot upright at all times are signals measured by the gyroscopes and accelerometers. These sensors provide information about various state variables at any given time.

The battery of the two-wheeled platform appears in Fig. 6 on the right side of the vehicle. However, in the realized physical system, the attachment of components (battery, electronics, etc.) will be located out in a way to assure uniform distribution of masses around the centre point of interaction of *x* and *y* axes.

### Advantages of using the proposed design with standard wheels over omnidirectional wheels

There are various types of wheels used in wheeled mobile robots including: standard, castor and omnidirectional wheels. The proposed design in this paper uses two standard wheels powered by two motors. The advantages of the used standard wheels include the simplicity in design and manufacturing and the relatively good reliability. The small size of used wheels (10 cm diameter) helps in providing better stability and stronger grip with the floor. This adds to the stability and rigidity of the entire system while carrying out material handling tasks. The simple manufacturing process of standard wheels assures minimum positioning errors while movement.

Omnidirectional wheels are used in mobile robots doing material handling tasks and other industrial applications, though mobile robots with omnidirectional wheels are controllable with reduced number of actuators and are highly manoeuvrable in narrow or crowded spaces. Accuracy of motion is influenced by systematic errors caused by unavoidable imperfections in the control and mechanical subsystems and nonsystematic caused by unpredictable phenomena such as wheel slippage and surface irregularities. Calibration will be needed to compensate for those errors due to the use of omnidirectional wheels. Other odometry errors, while the robot movement, may also exist due to unequal wheels diameters, joints misalignment, backlash and slippage in encoder pulses [24]. Omnidirectional vehicles are widely used in mobile robots for materials handling vehicles for logistics and wheelchairs. However, they are generally designed for the case of motion on flat, smooth terrain and are not feasible for outdoor usage [17]. Slippage is there when omnidirectional wheels are in motion and manufacturing of those wheels is an expensive and needs high accuracy. Furthermore, there is a poor efficiency because not all the wheels are rotating in the direction of movement, which causes loss from friction, and are more computationally complex because of the angle calculations of movement [25].

### Description of the system DOFs

**Fig. 8:**
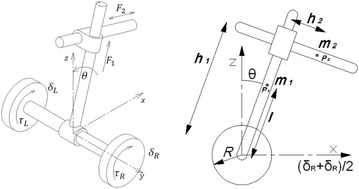
Schematic diagram of the system showing motion variables

**Fig. 9:**
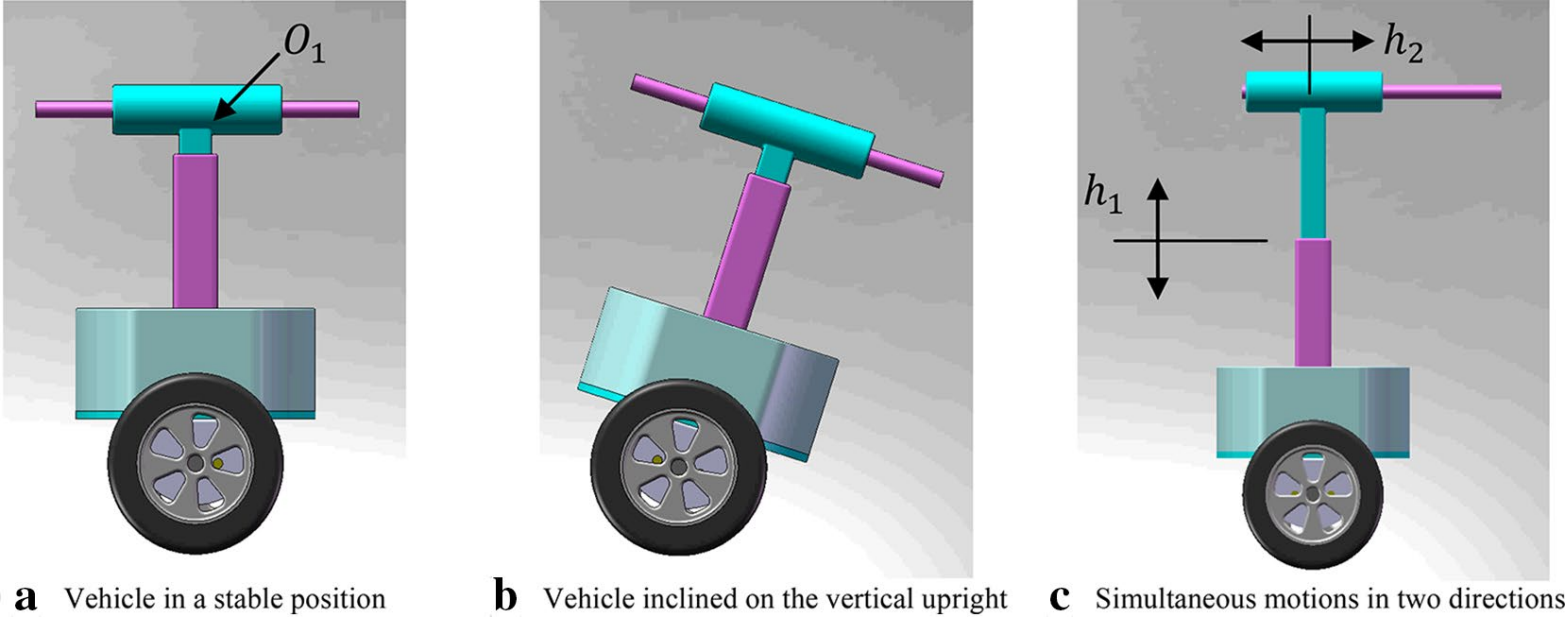
Mobility of the vehicle. **a** Vehicle position in the upright vertical, **b** inclination of the vehicle with respect to the upright position and **c** two linear motion of payload in two perpendicular axes

The considered system has degrees of freedom described by four types of translations with respect to the X and Z axes. They are represented by the angular displacement of the angular rotation of the right and left wheels $\delta_{\text{R}}$ and $\delta_{L}$, respectively, the attached payload linear displacement in vertical and horizontal directions $h_{1}$ and $h_{2}$, respectively, as shown in Fig. 8. The fifth DOF is represented by the tilt angle of the IB around the vertical Z axis, $\theta$. This configuration of the vehicle is believed to serve in various applications including but not limited to object picking and placing, as shown in Fig. 9, assembly lines and similar industrial and service robot applications that require working in tiny spaces.

**Fig. 10:**
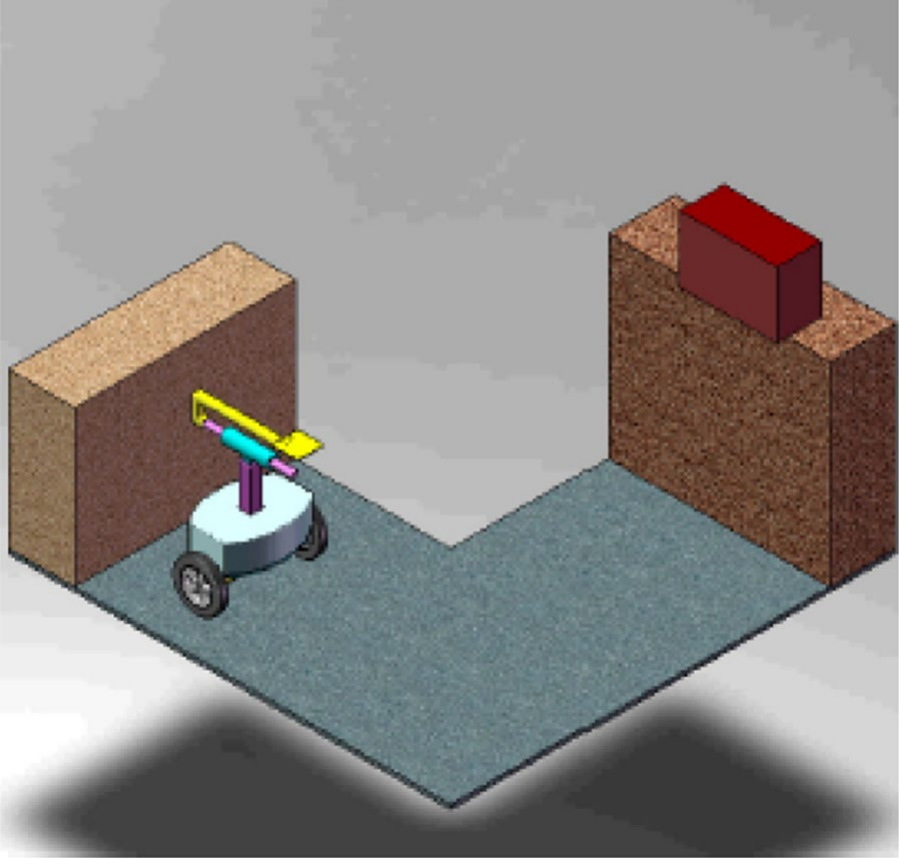
Example application: object pick and place

For the vehicle to undertake a picking and placing scenario shown in Fig. 10, the description of the course of motion can be explained as follows:

- The vehicle to start moving on two wheels while keeping a balance condition till reaching a desired location for picking the object. The dominant control efforts during this stage are the two control torque signals from the motors attached to the wheels.
- Once reaching a suitable position to pick the object, the linear actuators start to work by extending the IB up to the object position by a linear displacement, $h_{1}$. In this case, the centre of mass (COM) of the vehicle is moving up and the wheels motors must apply the torque necessary to keep a balance condition.
- Following the extension of the IB in a vertical position, the control system orders the linear actuator to extend the end-effector to extend in a lateral direction to the location of the object. As a consequence, the COM of the entire vehicle is changing its position and it is the responsibility of wheels motors to develop the appropriate motor torque that compensate for this change in the COM position. It is assumed in this stage of the research that the joint at $O_{1}$ is rigid and the two axes of motion for $h_{1}$ and $h_{2}$ are always perpendicular to each other. However, at further stage an active revolute joint should be used to ensure that the motion of $h_{2}$, to pick/place the object, is always in a horizontal direction. This is to reduce the change in the COM and hence to reduce the control effort required. While picking the object, the vehicle is expected to be subjected to sudden disturbance due to impact with the object. This should be overcome by the control signals from the wheels motors.
- Following picking up of the object, the end-effector should undergo a reverse motion back to its original position. This motion will be accompanied by re-adjustment of the COM again to its original position. The linear actuator should apply the appropriate force signal during this stage with the appropriate speed that makes the entire vehicle safe against tipping over. The vehicle needs to keep balancing depending on the torque signals developed by the wheels motors.
- As the rod of the linear actuator becomes in its original position, the IB begins travelling down to the desired height to place the object in the allocated place. The closer the COM to the chassis, the higher the control effort needs to be exerted by the motor wheels [13, 14].
- Finally, the end-effector extends till a desired location to place the object. This may include manoeuvring the entire vehicle to adjust the end-effector to do the task appropriately.
- Switching mechanisms need to be designed as a main part of the control algorithms to determine the sequence of engagement of each individual actuator associated with specific tasks in the above-mentioned stages.

Based on the above-mentioned motion description, Table 1 shows the engagement of each individual actuator against DOFs of the system during each of the substages during a picking and placing scenario of an object.

**Table 1:** Engagement of individual actuators against subtasks

Ser.	Subtask	DOFs associated	Right wheel motor $\tau_{R}$	Left wheel motor $\tau_{L}$	Linear actuator I $F_{1}$	Linear actuator II $F_{2}$
a	Moving till the picking place	$_{{\delta_{R} }}$, $_{{\delta_{L} }}$, $_{\theta }$	✓	✓	×	×
b	Extension of the IB	$_{{\delta_{R} }}$, $_{{\delta_{L} }}$, $_{\theta }$, $_{{h_{1} }}$	✓	✓	✓	×
c	Extension of the end-effector	$_{{\delta_{R} }}$, $_{{\delta_{L} }}$, $_{\theta }$, $_{{h_{2} }}$	✓	✓	×	✓
d	Reverse motion of the end-effector	$_{{\delta_{R} }}$, $_{{\delta_{L} }}$, $_{\theta }$, $_{{h_{2} }}$	✓	✓	×	✓
e	Contraction of the IB	$_{{\delta_{R} }}$, $_{{\delta_{L} }}$, $_{\theta }$, $_{{h_{1} }}$	✓	✓	✓	×
f	Placing of the object	$_{{\delta_{R} }}$, $_{{\delta_{L} }}$, $_{\theta }$, $_{{h_{2} }}$	✓	✓	×	×

As indicated in the table, the wheels motors are always engaged during the entire process as there are always a change in the location of the COM and possibility of external disturbance during the picking and/or placing of the object. For all subtasks, (a–f), the wheels motors need to develop the appropriate torque signal that is sufficient to keep the vehicle balance in an upright vertical position. The engagement of linear actuators will be as when needed during the picking, placing stages to complete both tasks. Switching mechanisms are designed to determine the period of engagement of each individual actuator.

## Mathematical modelling

The mathematical model of mechanical system is used to examine different behaviours of the model. In addition, it relates the kinematics of the mechanical system to the forces/torques applied to its links. The mathematical model of the proposed machine is generated in this section using the system physical parametric specifications that are shown in Table 2.

**Table 2:** Parameters and description

Parameter	Description	Value	Unit
$m_{1}$	Mass of the chassis	3.1	kg
$m_{2}$	Mass of the linear actuators	0.6	kg
$m_{\rm w}$	Mass of wheel	0.14	kg
$g$	Gravitational acceleration	9.81	m/s^2^
$l$	Distance of chassis’s centre of mass for wheel axle	0.14	m
$R$	Wheel radius	0.05	m
$J_{1}$	Rotation inertia of	0.068	kg m^2^
$J_{2}$	Rotation inertia of	0.0093	kg m^2^
$J_{\rm w}$	Rotation inertia of a wheel	0.000175	kg m^2^
$\mu_{c}$	Coefficient of friction between chassis and wheel	0.1	Ns/m
$\mu_{\rm w}$	Coefficient of friction between wheel and ground	0	Ns/m
$\mu_{1}$	Coefficient of friction of vertical linear actuator	0.3	Ns/m
$\mu_{2}$	Coefficient of friction of horizontal linear actuator	0.3	Ns/m

The friction at the mating surfaces has been simplified for the chassis–wheel, wheel–ground interaction and in the linear actuator to follow coulomb frictional model. The values of the coefficients have been selected depending on the type of surfaces. The selected constant values are assumed to be valid under all working conditions of the vehicle and the actuators. This did not take into account variations in speed, path configuration, terrain profile, etc. The constant values have been used to validate the system model. However, modelling interactions between surfaces need to be investigated for various surfaces, various terrain profiles and various operation conditions of the vehicle. The work done by Silva et al. [31] will be considered in future studies as suggested modelling technique for the wheel–ground interaction through modelling of foot–ground interaction of artificial locomotion systems.

### Deriving equations of motion

Based on the schematic diagram shown in Fig. 10, the linear displacement of the chassis COM, point $P_{1}$, can be derived as shown in Eqs. 1 and 2 along the X and Z axes, respectively, as follows:

1 $$ x_{1} = \left( {\frac{{\delta_{\text{R}} + \delta_{\text{L}} }}{2}} \right) + l\sin \theta $$

2 $$ z_{1} = l\cos \theta $$

where for the lateral linear displacement point $P_{2}$ can be calculated as follows:

3 $$ x_{2} = h_{1} \sin \theta + h_{2} \cos \theta + \left( {\frac{{\delta_{\text{R}} + \delta_{\text{L}} }}{2}} \right) $$

4 $$ z_{2} = h_{1} \cos \theta - h_{2} \sin \theta $$

#### Modelling using Lagrange formulation

Lagrange formulation is used in this section to derive model of the system since it provides a powerful technique for obtaining the equations of motion. The general form of Lagrange equation is identified as shown in Eq. 5.

5 $$ \frac{d}{{{\text{d}}t}}\left( {\frac{\partial L}{{\partial \dot{q}_{i} }}} \right) - \frac{\partial L}{{\partial q_{i} }} + \frac{\partial D}{{\partial \dot{q}_{i} }} = f_{i} $$

where *L* represents the Lagrange equation and it is determined as:

6 $$ L = T - U $$

where *T* and *U* are the total kinetic energies and potential energies of the system, respectively.

- $q_{i} \quad (i = 1,2, \ldots ,n)$ are generalized coordinates such as: $$ q_{i} = \left[ {\begin{array}{*{20}c} {h_{1} } & {h_{2} } & \theta & {\delta_{\text{L}} } & {\delta_{\text{R}} } \\ \end{array} } \right] $$
- $f_{i}$ is generalized forces that contain all the given forces in the system acting along the coordinates such as: $$ f_{i} = \left[ {\begin{array}{*{20}c} {F_{1} } & {F_{2} } & 0 & {\tau_{\text{L}} } & {\tau_{\text{R}} } \\ \end{array} } \right] $$
- $D$ is the dissipation function and illustrated as $D = \tfrac{1}{2}bq_{i}^{2}$

The total kinetic energy of the chassis can be calculated as follows:

7 $$ T_{c} = \tfrac{1}{2}m_{1} \left( {v_{x1}^{2} + v_{z1}^{2} } \right) + \tfrac{1}{2}m_{2} \left( {v_{x2}^{2} + v_{z2}^{2} } \right) + \tfrac{1}{2}J_{1} \dot{\theta }^{2} + \tfrac{1}{2}J_{2} \dot{\theta }^{2} $$

where

8 $$ v_{x1} = \tfrac{1}{2}v_{R} + \tfrac{1}{2}v_{L} + l\dot{\theta }\cos \theta $$

9 $$ v_{z1} = - l\dot{\theta }\sin \theta $$

10 $$ v_{x2} = \dot{h}_{1} \sin \theta + h_{1} \dot{\theta }\cos \theta + \dot{h}_{2} \cos \theta - h_{2} \dot{\theta }\sin \theta + \tfrac{1}{2}v_{R} + \tfrac{1}{2}v_{L} $$

11 $$ v_{z2} = \dot{h}_{1} \cos \theta - h_{1} \dot{\theta }\sin \theta - \dot{h}_{2} \sin \theta - h_{2} \dot{\theta }\cos \theta $$

The total kinetic energy per wheel can be calculated as follows:

12 $$ T_{w} = \tfrac{1}{2}m_{w} v_{R}^{2} + \tfrac{1}{2}m_{w} v_{L}^{2} + \tfrac{1}{2}J_{w} \left( {\frac{{v_{R}^{2} }}{{R^{2} }}} \right) + \tfrac{1}{2}J_{w} \left( {\frac{{v_{L}^{2} }}{{R^{2} }}} \right) $$

The total kinetic energy of the chassis and wheels can be calculated as follows:

13 $$ T = T_{\text{c}} + T_{\text{w}} $$

The total potential energy of the chassis and wheels can be calculated as follows:

14 $$ U = m_{1} gl\cos \theta + m_{2} g(h_{1} \cos \theta - h_{2} \sin \theta ) $$

The total dissipation energy of the chassis and wheels can be calculated as follows:

15 $$ D = \tfrac{1}{2}\mu_{1} \dot{h}_{1}^{2} + \tfrac{1}{2}\mu_{2} \dot{h}_{2}^{2} + \tfrac{1}{2}\mu_{w} \left( {\frac{{v_{\text{R}}^{2} + v_{\text{L}}^{2} }}{{R^{2} }}} \right) + \tfrac{1}{2}\mu_{\text{c}} \left( {v_{\text{R}}^{2} + v_{\text{L}}^{2} } \right) $$

Substituting Eqs. 13 and 14 in Eq. 6, the Lagrange equation can be expressed as follows:

16 $$ \begin{aligned} L & = \tfrac{1}{2}m_{1} \left( {v_{x1}^{2} + v_{z1}^{2} } \right) + \tfrac{1}{2}m_{2} \left( {v_{x2}^{2} + v_{z2}^{2} } \right) + \tfrac{1}{2}J_{1} \dot{\theta }^{2} + \tfrac{1}{2}J_{2} \dot{\theta }^{2} + \tfrac{1}{2}m_{w} \left( {v_{\text{R}}^{2} + v_{\text{L}}^{2} } \right) \\ & \quad + \tfrac{1}{2}J_{\text{w}} \left( {\frac{{v_{\text{R}}^{2} + v_{\text{L}}^{2} }}{{R^{2} }}} \right) - m_{1} gl\cos \theta - m_{2} g(h_{1} \cos \theta - h_{2} \sin \theta ) \\ \end{aligned} $$

Deriving the equation for $h_{1}$:

17 $$\tfrac{1}{2}m_{2} (2g\cos \theta - 2h_{1} \dot{\theta }^{2} - 4\dot{h}_{2} \dot{\theta } - 2h_{2} \ddot{\theta } + 2\textit{\"{h}}_{1} + (\ddot{\delta}_{R} + \ddot{\delta }_{L} )\sin \theta ) = F_{1} - \mu_{1} \dot{h}_{1}$$

Deriving the equation for $h_{2}$:

18 $$ \tfrac{1}{2}m_{2} (2g\sin \theta + 2h_{2} \dot{\theta }^{2} - 4\dot{h}_{1} \dot{\theta } - 2h_{1} \ddot{\theta } - 2\textit{\"{h}}_{2} - (\ddot{\delta }_{\text{R}} + \ddot{\delta }_{\text{L}} )\cos \theta ) = F_{2} - \mu_{2} \dot{h}_{2} $$

Deriving the equation for $\delta_{L}$:

19 $$ \begin{aligned} \tfrac{1}{2}m_{1} \left( {\tfrac{1}{2}\ddot{\delta }_{R} + \tfrac{1}{2}\ddot{\delta }_{L} - l\dot{\theta }^{2} \sin \theta + l\ddot{\theta }\cos \theta } \right) + \tfrac{1}{2}m_{2} \left( {\textit{\"{h}}_{1} \sin \theta + 2\dot{h}_{1} \dot{\theta }\cos \theta - h_{1} \dot{\theta }^{2} \sin \theta + h_{1} \ddot{\theta }\cos \theta } \right. \hfill \\ \left. {\quad + \textit{\"{h}}_{2} \cos \theta - 2\dot{h}_{2} \dot{\theta }\sin \theta - h_{2} \dot{\theta }^{2} \cos \theta - h_{2} \ddot{\theta }\sin \theta + \tfrac{1}{2}\ddot{\delta }_{R} + \tfrac{1}{2}\ddot{\delta }_{\text{L}} } \right) + 2m_{w} \ddot{\delta }_{\text{L}} + 2J_{\text{w}} \frac{{\ddot{\delta }_{L} }}{{R^{2} }} \hfill \\ = \tau_{L} - \mu_{\text{w}} \left( {\frac{{\dot{\delta }_{\text{L}} }}{{R^{2} }}} \right) - \mu_{c} \dot{\delta }_{L} \hfill \\ \end{aligned} $$

Deriving the equation for $\delta_{R}$:

20 $$ \begin{aligned} \tfrac{1}{2}m_{1} \left( {\tfrac{1}{2}\ddot{\delta }_{R} + \tfrac{1}{2}\ddot{\delta }_{L} - l\dot{\theta }^{2} \sin \theta + l\ddot{\theta }\cos \theta } \right) + \tfrac{1}{2}m_{2} \left( {\textit{\"{h}}_{1} \sin \theta + 2\dot{h}_{1} \dot{\theta }\cos \theta - h_{1} \dot{\theta }^{2} \sin \theta + h_{1} \ddot{\theta }\cos \theta } \right. \hfill \\ \left. { + \textit{\"{h}}_{2} \cos \theta - 2\dot{h}_{2} \dot{\theta }\sin \theta - h_{2} \dot{\theta }^{2} \cos \theta - h_{2} \ddot{\theta }\sin \theta + \tfrac{1}{2}\ddot{\delta }_{R} + \tfrac{1}{2}\ddot{\delta }_{L} } \right) + 2m_{w} \ddot{\delta }_{R} + 2J_{w} \frac{{\ddot{\delta }_{R} }}{{R^{2} }} \hfill \\ = \tau_{R} - \mu_{w} \left( {\frac{{\dot{\delta }_{R} }}{{R^{2} }}} \right) - \mu_{c} \dot{\delta }_{R} \hfill \\ \end{aligned} $$

Deriving the equation for $\theta$:

21 $$ \begin{aligned} 2m_{2} \dot{\theta }\left( {\dot{h}_{2} h_{2} + \dot{h}_{1} h_{1} } \right) + \tfrac{1}{2}m_{2} (h_{1} \cos \theta - h_{2} \sin \theta )\left( {\ddot{\delta }_{\text{R}} + \ddot{\delta }_{\text{L}} } \right) + \tfrac{1}{2}m_{1} l\cos \theta \left( {\ddot{\delta }_{\text{R}} + \ddot{\delta }_{\text{L}} } \right)   - m_{2} g(h_{1} \sin \theta + h_{2} \cos \theta ) + \ddot{\theta }(J_{1} + J_{2} + m_{1} l^{2} + m_{2} h_{2}^{2} + m_{2} h_{1}^{2} ) \hfill \\ +\, m_{2} \left( {\textit{\"{h}}_{2} h_{1} + \textit{\"{h}}_{1} h_{2} } \right) - m_{1} gl\sin \theta = 0 \hfill \\ \end{aligned} $$

Equations (17–21) represent the nonlinear second-order differential equations representing the dynamics of the system under consideration.

## State space modelling

In order to linearize the system, an equilibrium point is considered at the vertical upright position. This is applied when the tilt angle is approaching a zero value. The system equations of motion can be reformulated in the following forms:

22 $$ \tfrac{1}{2}m_{2} (2g - 4\dot{h}_{2} \dot{\theta } - 2h_{2} \ddot{\theta } + 2\textit{\"{h}}_{1} + \left( {\ddot{\delta }_{\text{R}} + \ddot{\delta }_{\text{L}} } \right)\theta ) = F_{1} - \mu_{1} \dot{h}_{1} $$

23 $$ \tfrac{1}{2}m_{2} \left( {2g\theta - 4\dot{h}_{1} \dot{\theta } - 2h_{1} \ddot{\theta } - 2\textit{\"{h}}_{2} - \ddot{\delta }_{R} - \ddot{\delta }_{L} } \right) = F_{2} - \mu_{2} \dot{h}_{2} $$

24 $$ \begin{aligned} \tfrac{1}{2}m_{1} \left( {\tfrac{1}{2}\ddot{\delta }_{\text{R}} + \tfrac{1}{2}\ddot{\delta }_{\text{L}} + l\ddot{\theta }} \right) + \tfrac{1}{2}m_{2} \left( {\textit{\"{h}}_{1} \theta + 2\dot{h}_{1} \dot{\theta } + h_{1} \ddot{\theta } + \textit{\"{h}}_{2} - 2\dot{h}_{2} \dot{\theta }\theta - h_{2} \ddot{\theta }\theta + \tfrac{1}{2}\ddot{\delta }_{R} + \tfrac{1}{2}\ddot{\delta }_{L} } \right) \hfill \\ \quad + 2m_{\text{w}} \ddot{\delta }_{\text{L}} + 2J_{\text{w}} \frac{{\ddot{\delta }_{\text{L}} }}{{R^{2} }} = \tau_{\text{L}} - \mu_{\text{w}} \left( {\frac{{\dot{\delta }_{\text{L}} }}{{R^{2} }}} \right) - \mu_{c} \dot{\delta }_{\text{L}} \hfill \\ \end{aligned} $$

25 $$ \begin{aligned} \tfrac{1}{2}m_{1} \left( {\tfrac{1}{2}\ddot{\delta }_{R} + \tfrac{1}{2}\ddot{\delta }_{L} + l\ddot{\theta }} \right) + \tfrac{1}{2}m_{2} \left( {\textit{\"{h}}_{1} \theta + 2\dot{h}_{1} \dot{\theta } + h_{1} \ddot{\theta } + \textit{\"{h}}_{2} - 2\dot{h}_{2} \dot{\theta }\theta - h_{2} \ddot{\theta }\theta + \tfrac{1}{2}\ddot{\delta}_{\text{R}} + \tfrac{1}{2}\ddot{\delta }_{\text{L}} } \right) \hfill \\ \quad + 2m_{\text{w}} \ddot{\delta }_{\text{R}} + 2J_{\text{w}} \frac{{\ddot{\delta }_{R} }}{{R^{2} }} = \tau_{\text{R}} - \mu_{\text{w}} \left( {\frac{{\dot{\delta }_{R} }}{{R^{2} }}} \right) - \mu_{\text{c}} \dot{\delta }_{\text{R}} \hfill \\ \end{aligned} $$

26 $$ \begin{aligned} 2m_{2} \dot{\theta }(\dot{h}_{2} h_{2} + \dot{h}_{1} h_{1} ) + \tfrac{1}{2}m_{2} (h_{1} - h_{2} \theta )\left( {\ddot{\delta }_{\text{R}} + \ddot{\delta }_{\text{L}} } \right) + \tfrac{1}{2}m_{1} l\left( {\ddot{\delta }_{\text{R}} + \ddot{\delta }_{\text{L}} } \right) - m_{2} g(h_{1} \theta + h_{2} ) \hfill \\ \quad + \ddot{\theta }\left( {J_{1} + J_{2} + m_{1} l^{2} + m_{2} h_{2}^{2} + m_{2} h_{1}^{2} } \right) + m_{2} \left( {\textit{\"{h}}_{2} h_{1} + \textit{\"{h}}_{1} h_{2} } \right) - m_{1} gl\theta = 0 \hfill \\ \end{aligned} $$

### State space modelling

The dynamics of the five DOFs machine can be represented by ten state vectors, *X* of the dynamic system as illustrated in the following equation:

27 $$ X = \left[ {\begin{array}{*{20}c} {\begin{array}{*{20}c} {\delta_{\text{R}} } & {\delta_{\text{L}} } & \theta & {h_{1} } & {h_{2} } \\ \end{array} } & {\begin{array}{*{20}c} {\dot{\delta }_{R} } & {\dot{\delta }_{L} } & {\dot{\theta }} & {\dot{h}_{1} } & {\dot{h}_{2} } \\ \end{array} } \\ \end{array} } \right] $$

where the state vector variables can be identified as follows:

- Right wheel displacement, $\delta_{\text{R}}$
- Left wheel displacement, $\delta_{\text{L}}$
- Chassis pitch angle, $\theta$
- Vertical linear link displacement, $h_{1}$
- Horizontal linear link displacement, $h_{2}$
- Right wheel velocity, $\dot{\delta }_{\text{R}}$
- Left wheel velocity, $\dot{\delta }_{\text{L}}$
- Chassis angular velocity, $\dot{\theta }$
- Vertical linear link velocity, $\dot{h}_{1}$
- Horizontal linear link velocity, $\dot{h}_{2}$

State variables of wheels velocity, angular velocity and linear velocities of the links are derivative of wheels displacements, links linear displacements and the pitch angle, respectively, and can be formulated as follows:

28 $$ X_{1} = \delta_{\text{R}} $$

29 $$ X_{2} = \delta_{\text{L}} $$

30 $$ X_{3} = \theta $$

31 $$ X_{4} = h_{1} $$

32 $$ X_{5} = h_{2} $$

33 $$ X_{6} = \dot{\delta }_{\text{R}} = \dot{X}_{1} $$

34 $$ X_{7} = \dot{\delta }_{\text{L}} = \dot{X}_{2} $$

35 $$ X_{8} = \dot{\theta } = \dot{X}_{3} $$

36 $$ X_{9} = \dot{h}_{1} = \dot{X}_{4} $$

37 $$ X_{10} = \dot{h}_{2} = \dot{X}_{5} $$

38 $$ \left[ {\begin{array}{*{20}c} {\dot{X}_{1} } \\ {\dot{X}_{2} } \\ {\dot{X}_{3} } \\ {\dot{X}_{4} } \\ {\dot{X}_{5} } \\ {\dot{X}_{6} } \\ {\dot{X}_{7} } \\ {\dot{X}_{8} } \\ {\dot{X}_{9} } \\ {\dot{X}_{10} } \\ \end{array} } \right] = \left[ {\begin{array}{*{20}c} {A_{11} } & {A_{12} } & {A_{13} } & {A_{14} } & {A_{15} } & {A_{16} } & {A_{17} } & {A_{18} } & {A_{19} } & {A_{110} } \\ {A_{21} } & {A_{22} } & {A_{23} } & {A_{24} } & {A_{25} } & {A_{26} } & {A_{27} } & {A_{28} } & {A_{29} } & {A_{210} } \\ {A_{31} } & {A_{32} } & {A_{33} } & {A_{34} } & {A_{35} } & {A_{36} } & {A_{37} } & {A_{38} } & {A_{39} } & {A_{310} } \\ {A_{41} } & {A_{42} } & {A_{43} } & {A_{44} } & {A_{45} } & {A_{46} } & {A_{47} } & {A_{48} } & {A_{49} } & {A_{410} } \\ {A_{51} } & {A_{52} } & {A_{53} } & {A_{54} } & {A_{55} } & {A_{56} } & {A_{57} } & {A_{58} } & {A_{59} } & {A_{510} } \\ {A_{61} } & {A_{62} } & {A_{63} } & {A_{64} } & {A_{65} } & {A_{66} } & {A_{67} } & {A_{68} } & {A_{69} } & {A_{610} } \\ {A_{71} } & {A_{72} } & {A_{73} } & {A_{74} } & {A_{75} } & {A_{76} } & {A_{77} } & {A_{78} } & {A_{79} } & {A_{710} } \\ {A_{81} } & {A_{82} } & {A_{83} } & {A_{84} } & {A_{85} } & {A_{86} } & {A_{87} } & {A_{88} } & {A_{89} } & {A_{810} } \\ {A_{91} } & {A_{92} } & {A_{93} } & {A_{94} } & {A_{95} } & {A_{96} } & {A_{97} } & {A_{98} } & {A_{99} } & {A_{910} } \\ {A_{101} } & {A_{102} } & {A_{103} } & {A_{104} } & {A_{105} } & {A_{106} } & {A_{107} } & {A_{108} } & {A_{109} } & {A_{1010} } \\ \end{array} } \right]\left[ {\begin{array}{*{20}c} {X_{1} } \\ {X_{2} } \\ {X_{3} } \\ {X_{4} } \\ {X_{5} } \\ {X_{6} } \\ {X_{7} } \\ {X_{8} } \\ {X_{9} } \\ {X_{10} } \\ \end{array} } \right] + \left[ {\begin{array}{*{20}c} {B_{11} } & {B_{12} } & {B_{13} } & {B_{14} } \\ {B_{21} } & {B_{22} } & {B_{23} } & {B_{24} } \\ {B_{31} } & {B_{32} } & {B_{33} } & {B_{34} } \\ {B_{41} } & {B_{42} } & {B_{43} } & {B_{44} } \\ {B_{51} } & {B_{52} } & {B_{53} } & {B_{54} } \\ {B_{61} } & {B_{62} } & {B_{63} } & {B_{64} } \\ {B_{71} } & {B_{72} } & {B_{73} } & {B_{74} } \\ {B_{81} } & {B_{82} } & {B_{83} } & {B_{84} } \\ {B_{91} } & {B_{92} } & {B_{93} } & {B_{94} } \\ {B_{101} } & {B_{102} } & {B_{103} } & {B_{104} } \\ \end{array} } \right]\left[ {\begin{array}{*{20}c} {u_{1} } \\ {u_{2} } \\ {u_{3} } \\ {u_{4} } \\ \end{array} } \right] $$

where

- $\tau_{\text{R}}$ and $\tau_{\text{R}}$ are the required torques for the right and left wheels,
- $F_{1}$ and $F_{1}$ are the generated linear force by the linear actuator for moving the payload in a vertical and horizontal direction, respectively.

where A, B, C and D matrices are shown as follows:

$$ A = \left[ {\begin{array}{*{20}c} 0 & 0 & 0 & 0 & 0 & 1 & 0 & 0 & 0 & 0 \\ 0 & 0 & 0 & 0 & 0 & 0 & 1 & 0 & 0 & 0 \\ 0 & 0 & 0 & 0 & 0 & 0 & 0 & 1 & 0 & 0 \\ 0 & 0 & 0 & 0 & 0 & 0 & 0 & 0 & 1 & 0 \\ 0 & 0 & 0 & 0 & 0 & 0 & 0 & 0 & 0 & 1 \\ 0 & 0 & {A_{63} } & 0 & {A_{65} } & {A_{66} } & {A_{67} } & 0 & 0 & {A_{610} } \\ 0 & 0 & {A_{73} } & 0 & {A_{75} } & {A_{76} } & {A_{77} } & 0 & 0 & {A_{710} } \\ 0 & 0 & {A_{83} } & 0 & {A_{85} } & {A_{86} } & {A_{87} } & 0 & 0 & {A_{810} } \\ 0 & 0 & 0 & {A_{94} } & 0 & 0 & 0 & 0 & 0 & 0 \\ 0 & 0 & {A_{103} } & 0 & {A_{105} } & {A_{106} } & {A_{107} } & 0 & 0 & {A_{1010} } \\ \end{array} } \right]\quad B = \left[ {\begin{array}{*{20}c} 0 & 0 & 0 & 0 \\ 0 & 0 & 0 & 0 \\ 0 & 0 & 0 & 0 \\ 0 & 0 & 0 & 0 \\ 0 & 0 & 0 & 0 \\ {B_{61} } & {B_{62} } & 0 & {B_{64} } \\ {B_{71} } & {B_{72} } & 0 & {B_{74} } \\ {B_{8} } & {B_{8} } & 0 & {B_{8} } \\ 0 & 0 & {B_{9} } & 0 \\ {B_{101} } & {B_{101} } & 0 & {B_{102} } \\ \end{array} } \right] $$

$$ C = \left[ {\begin{array}{*{20}c} 1 & 0 & 0 & 0 & 0 & 0 & 0 & 0 & 0 & 0 \\ 0 & 1 & 0 & 0 & 0 & 0 & 0 & 0 & 0 & 0 \\ 0 & 0 & 1 & 0 & 0 & 0 & 0 & 0 & 0 & 0 \\ 0 & 0 & 0 & 1 & 0 & 0 & 0 & 0 & 0 & 0 \\ 0 & 0 & 0 & 0 & 1 & 0 & 0 & 0 & 0 & 0 \\ \end{array} } \right]\quad D = \left[ {\begin{array}{*{20}c} 0 & 0 & 0 & 0 \\ 0 & 0 & 0 & 0 \\ 0 & 0 & 0 & 0 \\ 0 & 0 & 0 & 0 \\ 0 & 0 & 0 & 0 \\ \end{array} } \right] $$

And finally the state space model of the system can be formulated as follows:

39 $$ X = \left[ {\begin{array}{*{20}c} 0 & 0 & 0 & 0 & 0 & 1 & 0 & 0 & 0 & 0 \\ 0 & 0 & 0 & 0 & 0 & 0 & 1 & 0 & 0 & 0 \\ 0 & 0 & 0 & 0 & 0 & 0 & 0 & 1 & 0 & 0 \\ 0 & 0 & 0 & 0 & 0 & 0 & 0 & 0 & 1 & 0 \\ 0 & 0 & 0 & 0 & 0 & 0 & 0 & 0 & 0 & 1 \\ 0 & 0 & {A_{63} } & 0 & {A_{65} } & {A_{66} } & {A_{67} } & 0 & 0 & {A_{610} } \\ 0 & 0 & {A_{73} } & 0 & {A_{75} } & {A_{76} } & {A_{77} } & 0 & 0 & {A_{710} } \\ 0 & 0 & {A_{83} } & 0 & {A_{85} } & {A_{86} } & {A_{87} } & 0 & 0 & {A_{810} } \\ 0 & 0 & 0 & {A_{94} } & 0 & 0 & 0 & 0 & 0 & 0 \\ 0 & 0 & {A_{103} } & 0 & {A_{105} } & {A_{106} } & {A_{107} } & 0 & 0 & {A_{1010} } \\ \end{array} } \right]\left[ {\begin{array}{*{20}c} {\delta_{\text{R}} } \\ {\delta_{\text{L}} } \\ \theta \\ {h_{1} } \\ {h_{2} } \\ {\dot{\delta }_{\text{R}} } \\ {\dot{\delta }_{\text{L}} } \\ {\dot{\theta }} \\ {\dot{h}_{1} } \\ {\dot{h}_{2} } \\ \end{array} } \right] + \left[ {\begin{array}{*{20}c} 0 & 0 & 0 & 0 \\ 0 & 0 & 0 & 0 \\ 0 & 0 & 0 & 0 \\ 0 & 0 & 0 & 0 \\ 0 & 0 & 0 & 0 \\ {B_{61} } & {B_{62} } & 0 & {B_{64} } \\ {B_{71} } & {B_{72} } & 0 & {B_{74} } \\ {B_{81} } & {B_{82} } & 0 & {B_{84} } \\ 0 & 0 & {B_{93} } & 0 \\ {B_{101} } & {B_{102} } & 0 & {B_{104} } \\ \end{array} } \right]\left[ {\begin{array}{*{20}c} {\tau_{\text{R}} } \\ {\tau_{\text{L}} } \\ {F_{1} } \\ {F_{2} } \\ \end{array} } \right] $$

Constants A and B in Eqs. 38 and 39 are described in the "Appendix" at the end of the paper.

## Numerical simulation

### Open-loop system response

**Fig. 11:**
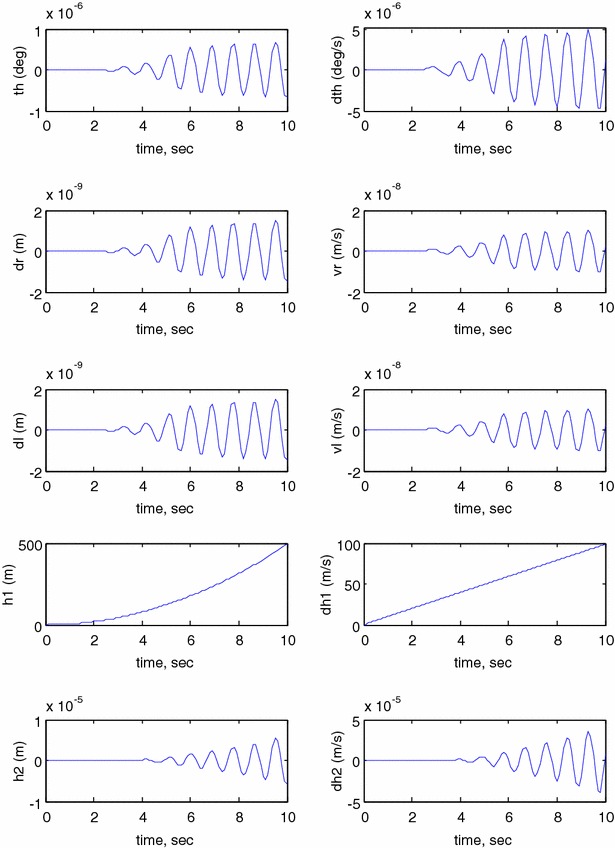
Open-loop system response

This section investigates the system responses and its performance using MATLAB and Simulink. In order to study the behaviour of the developed model, an open-loop system response has to be investigated. The model is simulated in MATLAB Simulink^®^ environment using the simulation parameters described in Table 2 where the following initial conditions are used: $\theta = 0$, $\delta_{\text{R}} = 0$, $\delta_{\text{L}} = 0$, $h_{1} = 0$, $h_{2} = 0$, $\dot{\theta } = 0$, $v_{\text{R}} = 0$, $v_{\text{L}} = 0$, $\dot{h}_{1} = 0$, $\dot{h}_{2} = 0$. Figure 11 illustrates the open-loop system response of pitch angle ($\theta$), angular velocity ($\dot{\theta }$), right wheel displacement ($\delta_{R}$), right wheel velocity ($v_{\text{R}}$), left wheel displacement ($\delta_{\text{L}}$), left wheel velocity ($v_{L}$), vertical link displacement ($h_{1}$), vertical link velocity ($\dot{h}_{1}$), horizontal link displacement ($h_{2}$) and horizontal link velocity ($\dot{h}_{2}$). As per the simulation results shown in Fig. 11, the system outputs reach infinity. It is clear that the system is unstable nonlinear system; therefore, a closed-loop system is required to stabilize the system and to improve its performance.

### Control scheme design

**Fig. 12:**
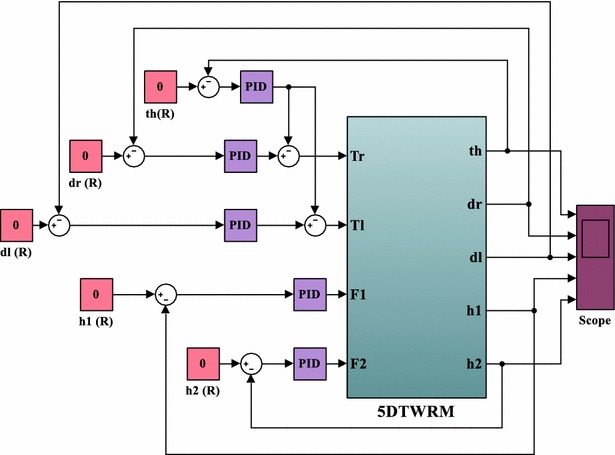
Schematic description of the control algorithm

The strategy to control the system depends on developing a feedback control mechanism of five control loops as shown in Fig. 12. In order to drive the vehicle to undergo a specific planar motion in the XY plane, two decoupled feedback loops are developed. The two feedback control loops occupy separate ranges of dynamics, low frequency and high frequency with tilt angle over higher frequency range and motion of intermediate body over lower frequency range, and hence, decoupling is reasonable to use and apply separate control loops. The input to both control loops is the error in the angular position of each wheel which measures the difference between the desired and actual angular positions of the corresponding wheel. The angular position of the IB is controlled by the measurement of the error in the tilt angle of the IB. In order to control the position of the object, two feedback control loops are developed with the error in the object position as an input and the actuation force as the output of the control loop. The inputs to the system are the driving torques of the wheels motors, $T_{\text{L}}$ and $T_{\text{R}}$, and the linear actuator forces, $F_{1}$ and $F_{2}$. The system has five outputs, the angular positions of the left and right wheels; $\delta_{\text{L}}$ and $\delta_{\text{R}}$, respectively, the angular positions of the IB, $\theta_{{}}$, and the linear displacements of the object, $h_{1}$ and $h_{2}$. The system is under-actuated by the virtue of having less actuation compared to number of system outputs. Five PID control loops are used to control the five outputs of the system. The control inputs are the error; Eqs. (40–44), the integral of the error and the derivative of error for the five measured variables, $\delta_{\text{L}}$, $\delta_{R}$, $\theta_{{}}$, $h_{1}$ and $h_{2}$, whereas the control outputs are the motor torques and the linear actuator forces.

40 $$ e_{{\delta_{\text{L}} }} = \delta_{\text{Ld}} - \theta_{\text{Lm}} $$

41 $$ e_{{\delta_{\text{R}} }} = \delta_{\text{Rd}} - \theta_{\text{Rm}} $$

42 $$ e_{\theta } = \theta_{\text{d}} - \theta_{\text{m}} $$

43 $$ e_{{h_{1} }} = h_{{1{\text{d}}}} - h_{{1{\text{m}}}} $$

44 $$ e_{{h_{2} }} = h_{{2{\text{d}}}} - h_{{2{\text{m}}}} $$

where *m* and *d* subscripts indicate desired and actual measured variable, respectively.

#### PID control without switching mechanisms

In the following simulation exercises, the developed control schemes are implemented on the system mathematical model identified in “Mathematical modelling” section. First, no switching mechanisms will be considered while running the simulation. The control algorithm and the system behaviour are tested in two various conditions, payload free motion and while considering the activation of the two linear actuators for both the horizontal and vertical motion of the payload. The same exercise is repeated after engaging switching mechanisms that are designed to determine when the linear actuators should start working.

#### Payload free movement (*h*_1_ = *h*_2_ = 0)

**Fig. 13:**
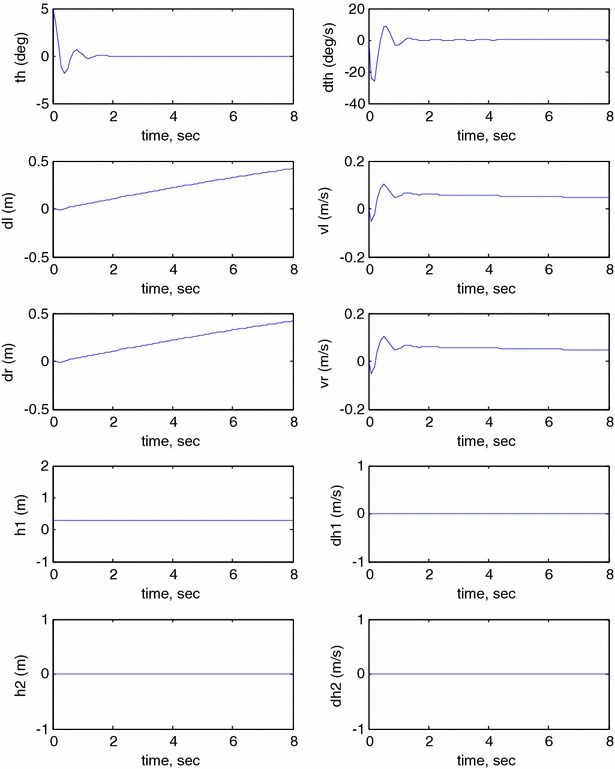
System output ($h_{1}$ = $h_{2}$ = 0), Unbounded wheels displacement

The behaviour of the robotic machine is observed for the rotation angle and velocity of the robot’s chassis, displacements and velocities of the two wheels, displacements and velocities of the linear actuators using different conditions as shown in the following figures. Figure 13 illustrates the output simulation of the system start initially at $\theta = 5^\circ$ and neglecting the effect of the linear actuators $h_{1}$ and $h_{2}$ by setting them to zero during the system stabilization.

**Fig. 14:**
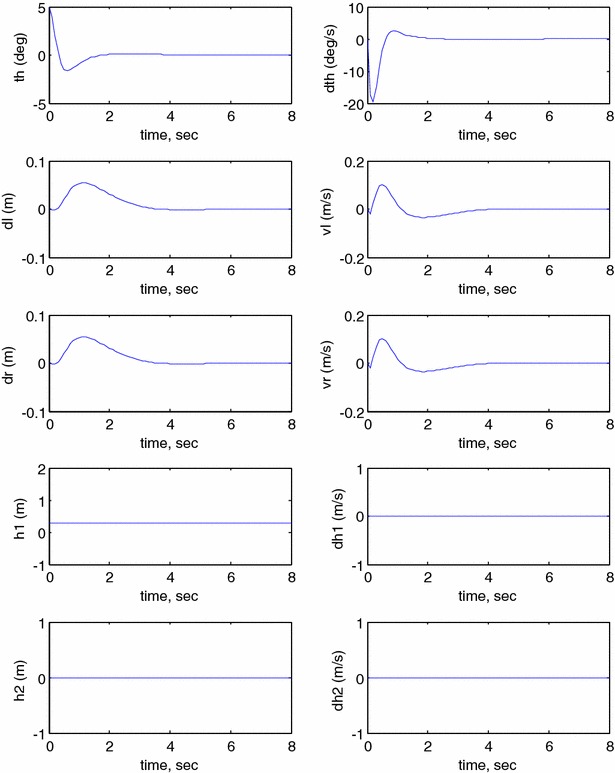
Modified system output ($h_{1}$ = $h_{2}$ = 0). Bounded wheels displacement

It can be noticed from Fig. 13 that the control mechanism stabilizes the vehicle to reach the balancing position in less than 2 s. However, the vehicle motion is unbounded and keeps moving in order to preserve the stability condition. This is considered as an undesirable behaviour; specifically, these types of vehicles are supposed to serve in minimum working space. The vehicle is considered to move with a fixed velocity once it achieves a stable position. In order to minimize the motion of the system, the controller is modified by bounding the linear displacement of the wheels as illustrated in Fig. 14. The wheels are allowed to rotate a pre-specified fraction which is equivalent to a boundary limit of 5-cm linear displacement. The control scheme is able to achieve the balancing position within 2 s, and the steady-state position of the wheels is reached within 4 s. Bounding the wheels rotation has a positive impact on the stabilization of the vehicle with limited disturbance compared to the previous case and hence less interruption in the control torques by the wheels motors.

#### Simultaneous horizontal and vertical motion (*h*_1_ and *h*_2_ ≠ 0)

**Fig. 15:**
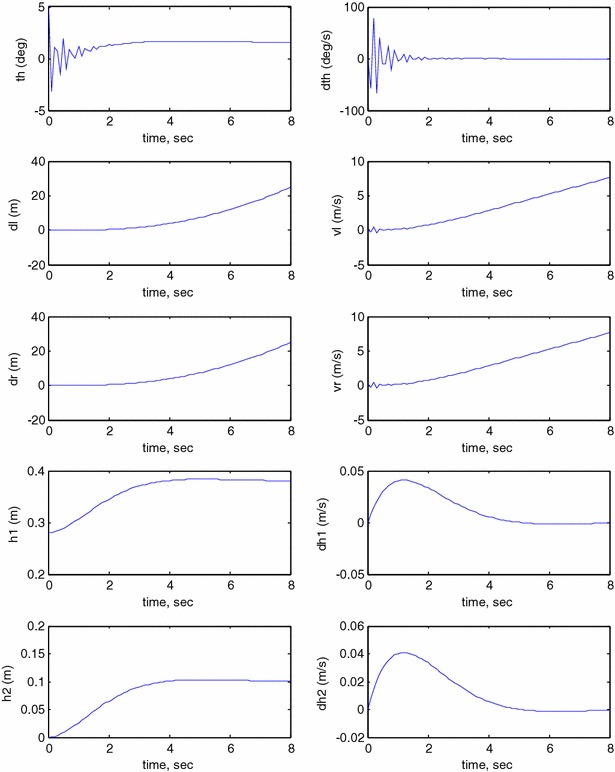
System output ($h_{1}$ and $h_{2}$ $\ne$ 0). Unbounded wheels displacement

This study investigates the impact of changing the COM of the vehicle in two mutually perpendicular axes. In this exercise, the linear actuators start to work by extending, simultaneously, in two perpendicular axes without considering a payload. As shown in Fig. 15, the system underwent through a longer transient period if compared to previous case ($h_{1}$ = $h_{2}$ = 0). It took longer for the system to reach a stable region; the overshoot is increased dramatically due to the change of the position of the COM in two different directions. The period taken by the vehicle to reach the stable range, around 4 s, is equivalent to the time taken by the linear actuator to extend in both axes, $h_{1}$ and $h_{2}$. The torques of the wheels motors are expected to be affected by such long transient period of the IB till reaching stability. Compared to previous simulation exercises, there has been large amount of vibration during the period of changing the COM in the two directions and this in turn will lead to changes in the control effort required.

### Design of switching mechanisms

**Fig. 16:**
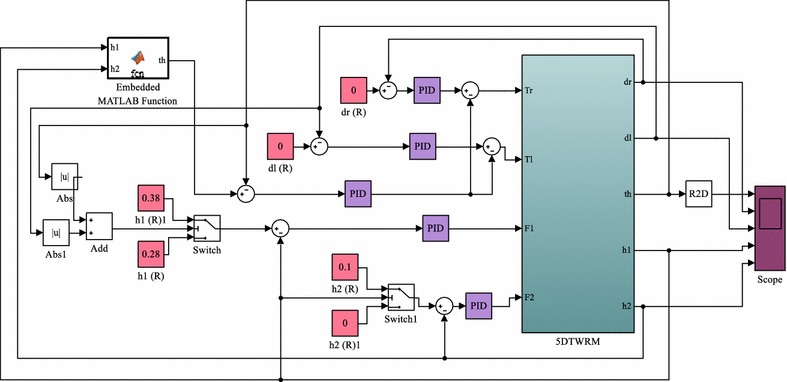
Modified control algorithm with switching mechanisms

Since the proposed platform is mainly designed for picking and/or placing applications, it is desirable to stabilize the system first. The reason is to avoid any disturbance at the start of working as a result of lifting an object. Lifting an object will result in moving the COM during the stabilization mode, and this in turn will affect the stability condition and disturbs the control effort. To avoid such situation, the control scheme is modified as illustrated in Fig. 16. Two switching mechanisms are added to the system to assure system stability before starting the object handling. The two mechanisms are developed in a way that the linear actuators will not activate unless the IB of the vehicle reaches the stable upright position.

Three case studies are considered where only one linear actuator is allowed to work at a time in the first two cases and then simultaneously working of the two actuators in the thirds case. Two signals are developed for both $h_{1}$ and $h_{2}$ using a signal builder block in MATLAB Simulink^®^.

#### Payload vertical movement only

**Fig. 17:**
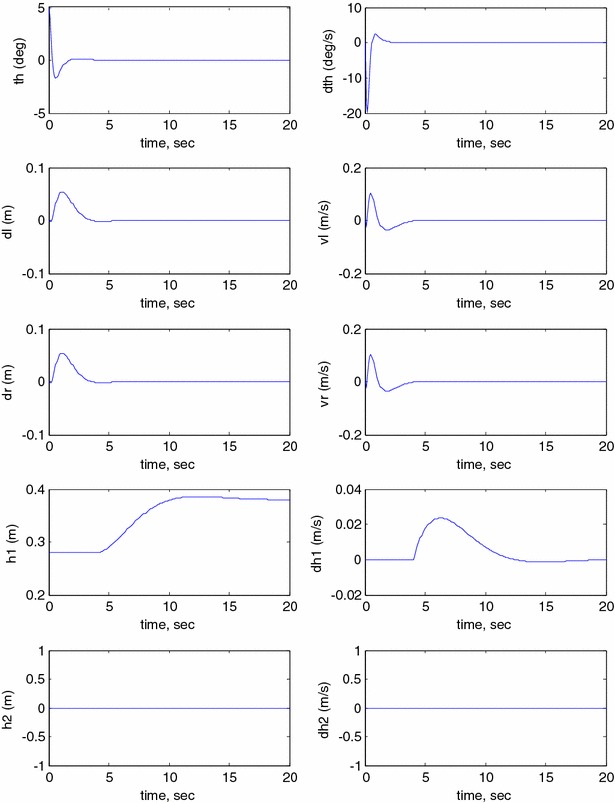
System output, payload vertical motion only

In this case, the linear actuator along the IB is allowed to work by moving up and down along the IB and z axis. This is physically implementing by extending and contraction of the linear actuator rod which leads to move the entire COM up and down as per the control signal developed by the actuator. Figure 17 illustrates the output simulation of the system that starts with initial conditions at $\theta = 5^\circ$, $h_{1} = 0.28$ *m* and $h_{2} = 0$. The actuator starts to extend to nearly 0.4 *m* after 5 s from the start of the simulation. The control mechanism was robust enough the way that no interruption occurred in the stabilization condition of the IB. The linear actuator accelerates to its maximum speed at around 7 s and then decelerates to settle down completely when reaching its desired height.

#### Payload horizontal movement only

**Fig. 18:**
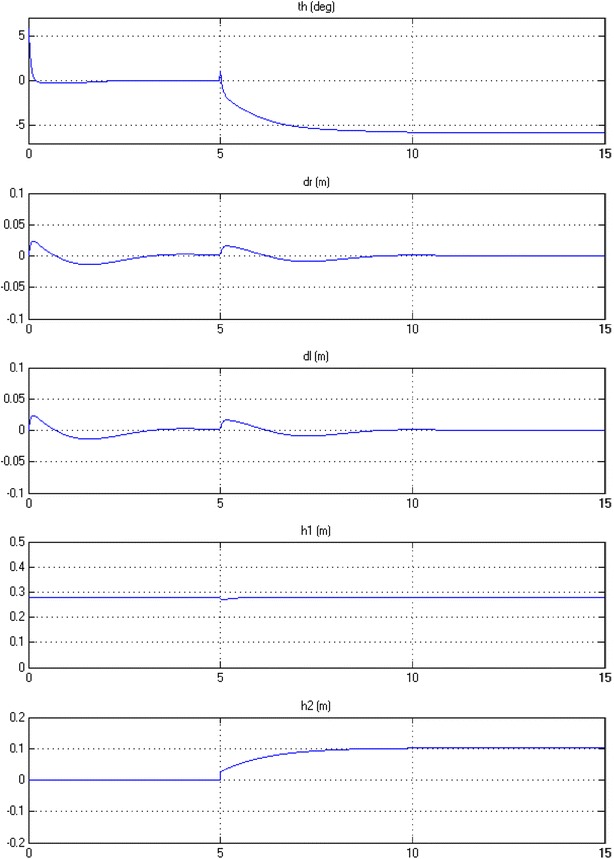
System output. Payload horizontal motion only

During this case, the system is simulated to observe the impact of changing $h_{2}$ in a direction perpendicular to the axis of the IB and in the *x* direction. This situation is similar to inclination of the IB forward or back and also simulates scenarios of wheeled machines moving up or down an inclined surface. The initial conditions are set as $\theta = 5^\circ$, $h_{1} = 0.28$ and $h_{2} = 0$. The actuator along the IB is kept locked during this stage, and the motion allowed will be the one from the other linear actuator who starts to work after achieving a balance condition as shown in Fig. 18. As observed from the figure, changing $h_{2}$ by only 10 cm at 5 s will act as a sudden impact disturbance which hits the IB causing it to change its direction dramatically to the opposite side of Z axis as obvious from the tilt angle graph. However, the control algorithm was not able to bring the IB to the vertical position and instead kept it inclined in the opposite side with a constant inclination angle of around 7°. Changing $h_{2}$ in the mentioned manner also has an impact on the linear motion of the vehicle in the X direction as clear from the fraction displacements of both wheels.

#### Payload simultaneous horizontal and vertical movements

**Fig. 19:**
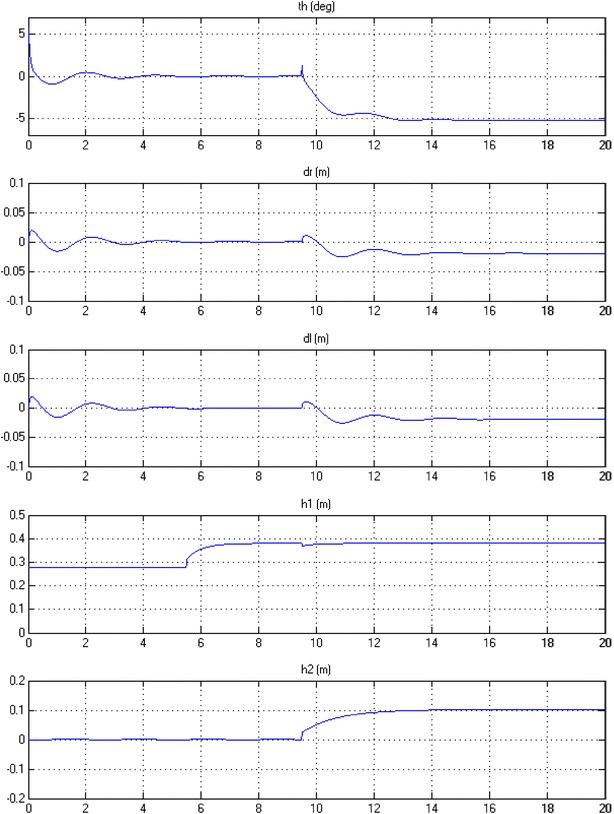
System output

In order to test the robustness of the proposed control algorithm, the system is simulated to observe the impact of changing $h_{1}$ and $h_{2}$ sequentially. $h_{1}$ is kept fixed at 0.28 cm for around (5.5) seconds before starting to change to its desired height. As expected and demonstrated earlier in Fig. 17, no interruption occurred in the stabilization condition of the IB. Changing $h_{2}$ starts at (9.5) seconds resulting in sudden changes in the stabilization of the IB and a slight disturbance in $h_{1}$. In response to the changes in $h_{2}$, the IB leans in opposite direction to compensate for the change in the position of the COM due to extension of $h_{2}$ as shown in Fig. 19.

The implementation of switching mechanism in the control algorithms has reflection on the simulation results and the way the system performs. This can be summarized as follows:

- Checking the robustness of the developed control approach. In Fig. 19, the IB leans in the opposite direction to compensate for the change in the position of the COM due to extension of $h_{2}$. Activating each individual actuator at a certain time tends to act as a sudden disturbance, in particular changing $h_{2}$ to the system which already achieved a stability.
- Adding switching helps the author to conclude that changing does not have significant impact on the output of the system as noticed in Figs. 17 and 19.
- Adding switching mechanisms mimics real scenarios in practical applications where not all actuators work at the same time.

The decoupled feedback control is believed that it is not related to the nonsmooth trajectories in Figs. 17 and 19. However, the fluctuations are due to the actuations of the linear actuators either simultaneously or consecutively. Further smoothness of the trajectory tracking can be achieved by minimizing the flexible dynamics items of the change in the tilt angle.

## Conclusions

A novel 5 DOFs two-wheeled machine is proposed in this work where the mathematical model is derived using Lagrangian dynamics. Dissipation energies are included in the system model for better consideration of nonlinear parameters. The configuration of the machine allows handling of an object in two mutually perpendicular directions that increase the workspace of current available configuration of TWMs. However, this will also be accompanied by situations where balance will be more complicated due to the change of the vehicle COM in different directions. The control of the vehicle will also become more complicated as a result of adding one more DOF to the system. Future considerations of this work will include but not limited to the following:

- Testing the vehicle in confined space for path tracking and picking and placing an object, this will include consideration of additional weight of an object, tracking a pre-specified trajectory, picking and placing the object from a certain location, carrying it and placing in desired location.
- Further investigation will include also workspace and kinematics analysis of the vehicle.
- Implementation of various optimization tools including bacterial forging (BF), spiral dynamics (SD) and hybrid spiral dynamics bacterial chemotaxis (HSDBC) for better performance of the system and improved energy consumption.
- Further investigation of the linear model of the system will be carried out while implementing various control approaches including fuzzy logic control (FLC).

## Appendix: Coefficients of the state space model

$$ A_{63} = \left[ {\frac{{4gR^{2} \left( {J_{w} + M_{w} R^{2} } \right)\left( {m_{2} \left( {m_{1} l^{2} + J_{1} + J_{2} } \right) + \left( {m_{1}^{2} l^{2} } \right)} \right)}}{{{\text{den}}_{1} }}} \right] $$

$$ A_{65} = \left[ {\frac{{4m_{1} m_{2} glR^{2} \left( {J_{\text{w}} + M_{\text{w}} R^{2} } \right)}}{{{\text{den}}_{1} }}} \right] $$

$$ A_{66} = \left[ {\frac{{8\left( {m_{1} l^{2} + J_{1} + J_{2} } \right)\left( {(J_{\text{w}} + M_{\text{w}} R^{2} )(\mu_{\text{w}} + \mu_{\text{c}} R^{2} )} \right) + m_{1} R^{2} \left( {J_{1} + J_{2} } \right)\left( {\mu_{\text{w}} + \mu_{\text{c}} R^{2} } \right)}}{{{\text{den}}_{1} }}} \right] $$

$$ A_{67} = - \left[ {\frac{{m_{1} R^{2} \left( {J_{1} + J_{2} } \right)\left( {\mu_{\text{w}} + \mu_{\text{c}} R^{2} } \right)}}{{{\text{den}}_{1} }}} \right], \quad A_{610} = \left[ {\frac{{\left( {4\mu_{2} R^{2} } \right)\left( {m_{1} l^{2} + J_{1} + J_{2} } \right)\left( {J_{\text{w}} + M_{\text{w}} R^{2} } \right)}}{{{\text{den}}_{1} }}} \right] $$

$$ B_{61} = - \left[ {\frac{{8R^{2} \left( {m_{1} l^{2} + J_{1} + J_{2} } \right)\left( {J_{\text{w}} + M_{\text{w}} R^{2} } \right) + m_{1} R^{4} \left( {J_{1} + J_{2} } \right)}}{{{\text{den}}_{1} }}} \right],B_{62} = \left[ {\frac{{m_{1} R^{4} (J_{1} + J_{2} )}}{{{\text{den}}_{1} }}} \right] $$

$$ B_{64} = - \left[ {\frac{{4R^{2} \left( {m_{1} l^{2} + J_{1} + J_{2} } \right)\left( {J_{\text{w}} + M_{\text{w}} R^{2} } \right)}}{{{\text{den}}_{1} }}} \right] $$

$$ A_{73} = A_{63}, \quad A_{75} = A_{65}, \quad A_{76} = A_{67}, \quad A_{710} = A_{610}, \quad B_{71} = B_{62}, \quad B_{72} = B_{61}, \quad B_{74} = B_{64}$$

$$ A_{83} = \left[ {\frac{{m_{1} gl\left( {4J_{\text{w}} + R^{2} \left( {4M_{\text{w}} + m_{1} + m_{2} } \right)} \right)}}{{{\text{den}}_{2} }}} \right] $$

$$ A_{85} = \left[ {\frac{{m_{2} g\left( {4J_{\text{w}} + 4M_{\text{w}} R^{2} + m_{1} R^{2} } \right)}}{{{\text{den}}_{2} }}} \right], \quad A_{86} = \left[ {\frac{{m_{1} l\mu_{\text{c}} R^{2} + m_{1} l\mu_{\text{w}} }}{{{\text{den}}_{2} }}} \right], \quad A_{87} = \left[ {\frac{{m_{1} l\mu_{\text{c}} R^{2} + m_{1} l\mu_{\text{w}} }}{{{\text{den}}_{2} }}} \right] $$

$$ A_{810} = \left[ {\frac{{m_{1} l\mu_{2} R^{2} }}{{{\text{den}}_{2} }}} \right], \quad B_{8} = - \left[ {\frac{{m_{1} lR^{2} }}{{{\text{den}}_{2} }}} \right],\quad A_{94} = \left[ { - \frac{{\mu_{1} }}{{m_{2} }}} \right], \quad B_{9} = \left[ {\frac{1}{{m_{2} }}} \right],\quad F_{11} = \left( {F_{1} - m_{2} g} \right) $$

$$ A_{103} = \left[ {\frac{{m_{2} g\left( {J_{1} + J_{2} + m_{1} l^{2} } \right)\left( {4J_{\text{w}} + 4M_{\text{w}} R^{2} + m_{1} R^{2} + m_{2} R^{2} } \right)}}{{{\text{den}}_{3} }}} \right], \quad A_{105} = \left[ {\frac{{m_{1} g_{{}} l_{{}} m_{2}^{2} R^{2} }}{{{\text{den}}_{3} }}} \right], $$

$$ A_{106} = \left[ {\frac{{m_{2} \left( {m_{1} l^{2} + J_{1} + J_{2} } \right)\left( {\mu_{\text{w}} + R^{2} \mu_{\text{c}} } \right)}}{{{\text{den}}_{3} }}} \right], \quad A_{107} = \left[ {\frac{{m_{2} \left( {m_{1} l^{2} + J_{1} + J_{2} } \right)\left( {\mu_{\text{w}} + R^{2} \mu_{\text{c}} } \right)}}{{{\text{den}}_{3} }}} \right] $$

$$ A_{1010} = \left[ {\frac{{\mu_{2} \left( {\left( {J_{1} + J_{2} + m_{1} l^{2} } \right)\left( {4J_{\text{w}} + 4M_{\text{w}} R^{2} + m_{2} R^{2} } \right) + \left( {J_{1} + J_{2} } \right)\left( {m_{1} R^{2} } \right)} \right)}}{{{\text{den}}_{3} }}} \right] $$

$$ B_{101} = - \left[ {\frac{{m_{2} R^{2} \left( {m_{1} l^{2} + J_{1} + J_{2} } \right)}}{{{\text{den}}_{3} }}} \right] $$

$$ B_{102} = - \left[ {\frac{{\left( {J_{1} + J_{2} + m_{1} l^{2} } \right)\left( {4J_{\text{w}} + 4M_{\text{w}} R^{2} + m_{2} R^{2} } \right) + \left( {J_{1} + J_{2} } \right)\left( {m_{1} R^{2} } \right)}}{{{\text{den}}_{3} }}} \right] $$

where

$$ \begin{aligned} {\text{den}}_{1} = - 4\left[ {4\left( {J_{1} + J_{2} + m_{1} l^{2} } \right)\left( {J_{\text{w}}^{2} + M_{\text{w}}^{2} R^{4} + 2J_{\text{w}} M_{\text{w}} R^{2} } \right) + \left( {J_{1} + J_{2} } \right)\left( {m_{1} M_{\text{w}} R^{4} + m_{1} J_{\text{w}} R^{2} } \right)} \right] \hfill \\ {\text{den}}_{2} = \left[ {4\left( {J_{1} + J_{2} + m_{1} l^{2} } \right)\left( {J_{\text{w}} + M_{\text{w}} R^{2} } \right)} \right] + \left[ {\left( {J_{1} + J_{2} } \right)\left( {m_{1} R^{2} } \right)} \right] \hfill \\ {\text{den}}_{3} = m_{2} \left[ {4\left( {J_{1} + J_{2} + m_{1} l^{2} } \right)\left( {J_{\text{w}} + M_{\text{w}} R^{2} } \right) + \left( {J_{1} + J_{2} } \right)\left( {m_{1} R^{2} } \right)} \right] \hfill \\ \end{aligned} $$
